# Population Differences in Transcript-Regulator Expression Quantitative Trait Loci

**DOI:** 10.1371/journal.pone.0034286

**Published:** 2012-03-27

**Authors:** Pierre R. Bushel, Ray McGovern, Liwen Liu, Oliver Hofmann, Ahsan Huda, Jun Lu, Winston Hide, Xihong Lin

**Affiliations:** 1 Biostatistics Branch, National Institute of Environmental Health Sciences, Research Triangle Park, North Carolina, United States of America; 2 Microarray and Genome Informatics, National Institute of Environmental Health Sciences, Research Triangle Park, North Carolina, United States of America; 3 Microarray Core, National Institute of Environmental Health Sciences, Research Triangle Park, North Carolina, United States of America; 4 Bioinformatics Core, Harvard School of Public Health, Boston, Massachusetts, United States of America; 5 Department of Biostatistics, Harvard School of Public Health, Boston, Massachusetts, United States of America; 6 SRA International Inc., Research Triangle Park, North Carolina, United States of America; University of California, United States of America

## Abstract

Gene expression quantitative trait loci (eQTL) are useful for identifying single nucleotide polymorphisms (SNPs) associated with diseases. At times, a genetic variant may be associated with a master regulator involved in the manifestation of a disease. The downstream target genes of the master regulator are typically co-expressed and share biological function. Therefore, it is practical to screen for eQTLs by identifying SNPs associated with the targets of a transcript-regulator (TR). We used a multivariate regression with the gene expression of known targets of TRs and SNPs to identify TReQTLs in European (CEU) and African (YRI) HapMap populations. A nominal *p*-value of <1×10^−6^ revealed 234 SNPs in CEU and 154 in YRI as TReQTLs. These represent 36 independent (tag) SNPs in CEU and 39 in YRI affecting the downstream targets of 25 and 36 TRs respectively. At a false discovery rate (FDR) = 45%, one *cis*-acting tag SNP (within 1 kb of a gene) in each population was identified as a TReQTL. In CEU, the SNP (rs16858621) in *Pcnxl2* was found to be associated with the genes regulated by CREM whereas in YRI, the SNP (rs16909324) was linked to the targets of miRNA hsa-miR-125a. To infer the pathways that regulate expression, we ranked TReQTLs by connectivity within the structure of biological process subtrees. One TReQTL SNP (rs3790904) in CEU maps to *Lphn2* and is associated (nominal *p*-value = 8.1×10^−7^) with the targets of the X-linked breast cancer suppressor Foxp3. The structure of the biological process subtree and a gene interaction network of the TReQTL revealed that tumor necrosis factor, NF-kappaB and variants in G-protein coupled receptors signaling may play a central role as communicators in Foxp3 functional regulation. The potential pleiotropic effect of the Foxp3 TReQTLs was gleaned from integrating mRNA-Seq data and SNP-set enrichment into the analysis.

## Introduction

Phenotypic differences between populations have been shown to be associated with variation in genes, the epigenome, the environment and quantitative traits. Gene expression has been used as a quantitative phenotypic trait to locate regions in the genome that have polymorphisms governing differential transcription within populations [1], [2], [3], [4]. This type of inference termed expression quantitative trait loci (eQTL) analysis has been used in genome-wide association studies (GWAS) to map single nucleotide polymorphisms (SNPs) to regions that affect gene expression [5]. Recently it has been shown that SNPs associated with a phenotypic trait are more likely to be eQTLs [6]. The advantage of understanding the contribution of genetic variations on the expression of genes has major implications on the manner in which pharmaceuticals are personalized for an individual and how complex diseases are investigated.

**Figure 1:**
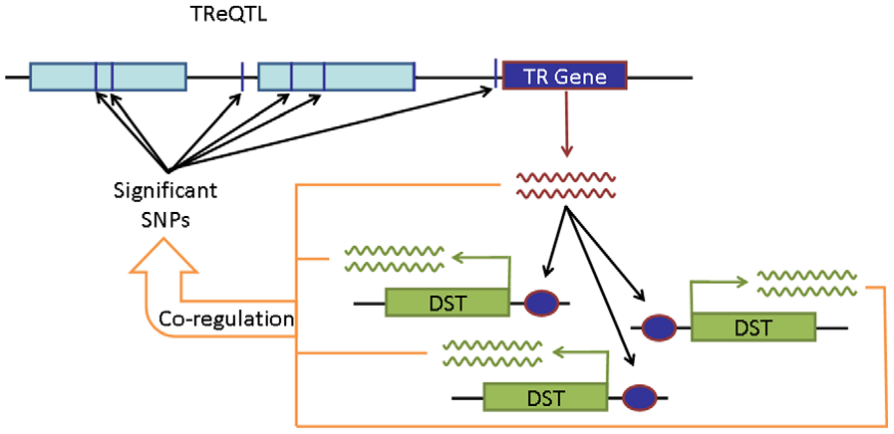
Strategy to identify transcript-regulator eQTLs (TReQTLs). The gene expression of downstream targets (DSTs) of a transcript-regulator (TR) is used as quantitative traits to associate with individual single nucleotide polymorphisms (SNPs). In some cases the SNPs map to the same gene, different genes, the TR or are intergenic.

A typical eQTL approach entails modeling the expression of a single gene as a response variable with the genotypes of a single SNP as the predictor variable. Variants of eQTL modeling take the form of a pathway, network component, sparse factor, cluster or the average of a group of co-expressed genes as the response variable and/or predict the expression according to a set of SNPs selected by LASSO, canonical correlation or interval mapping [7], [8], [9], [10], [11], [12], [13], [14]. The goal is to determine if there are “eQTL hotspots” [15] where a SNP leads to widespread changes in the expression of genes that are coordinately regulated. Hallmark examples of the power of eQTL analysis for determination of population differences are illustrated by several recent bodies of work. For example, several investigators have demonstrated the robustness of eQTLs to discern variation in gene expression between populations due to environmental exposures or geographic ancestry [16], [17], [18], [19]. Others have shown that gene expression can vary according to particular genotypes, chemical agents and factors such as tissue type, gender, genotype and age [20], [21], [22], [23], [24], [25]. Also, many have successfully linked genetic variants to transcriptional patterns within ethnic groups [26] although batch effects and biological noise confounding the differences between the populations can distort the interpretation of the results [27], [28]. For instance, the SCAN database [29] is a catalogue of the association of a given SNP to variations in gene expression between Yoruba in Ibadan, Nigeria (African: YRI) and CEPH-Utah residents with ancestry from northern and western Europe (European: CEU) HapMap populations [30]. These variants can be *cis*- or *trans*-acting whereby the effect is situated proximal to the expressed gene or it is located elsewhere in the genome, respectively. Although it has been suggested that SNPs residing in transcription factors (TFs) have no significant attributable effect on gene expression variation [11], it is unknown whether a variant that affects the genes regulated by a TF operates through a system of regulated pathways. Therefore, a more comprehensive way to better understand the genetic component of variation in gene expression within and between populations is to address the problem on a systems biology level. In other words, on a genome-wide scale, simultaneously model the expression of genes that are downstream targets (DSTs) of a transcript-regulator (TR) (Figure 1). A TR can be a TF, a cofactor, a complex, a microRNA or combination of these which are involved in the regulation of transcription and govern signaling pathways.

Li et al. [9] demonstrated the practicality of utilizing pathways as a response variable to associate SNPs between two genotype categories. They identified several genome-wide pathway regulators that seem to mediate gene expression differences. The advantages of this type of TReQTL approach are that the covariance of the DSTs is used in the modeling, co-regulation of the genes is inferred and the eQTL scan is genome-wide. Hence, scanning for TReQTLs is more likely to reveal variants that confer differences in gene expression between populations through genetically-wired regulatory mechanisms. We used a multivariate linear regression to model the DSTs of TRs with SNPs typed in CEU and YRI populations. The DSTs of the TRs were obtained from the TRANSFAC® knowledgebase - a biological resource that catalogs experimentally-proven binding and regulation of genes by various TRs [31], [32]. For all TRs with two or more DSTs, we detected 234 SNPs in CEU and 154 in YRI representing 36 and 39 independent (tag) SNPs as TReQTLs and affecting the DSTs of 25 and 36 TRs respectively. The expression of the DSTs of 24 TRs was associated with SNPs in both populations. Mapping to within 1 kb of a gene and controlling for multiple testing revealed one *cis*-acting tag SNP in each population as a TReQTL. In CEU, a TReQTL SNP was found to be associated with the DSTs of the X-linked breast cancer suppressor Foxp3 but is not significant in the YRI dataset. The Foxp3 TReQTL SNPs were overrepresented in evolutionary conserved regions (ECRs) of the genome in CEU and enriched in splice junctions (SJs) in YRI.

## Results

### Analysis Strategy

Typical expression quantitative trait loci (eQTL) analyses take the form of a strategy where a single gene is used as a response variable and individual single nucleotide polymorphisms (SNPs) the predictor variable to determine if there is association of a particular phenotype with a variant. The correlation of co-regulated genes is not taken into consideration. We used a multivariate approach to leverage the covariance of the gene expression of downstream targets (DSTs) of a transcript-regulator (TR) to perform genome-wide associations for SNPs that are potentially linked to changes in gene expression across genotypes. The genotype data (phase-II, release 24, forward strand, non-redundant) from the 60 Yoruba in Ibadan, Nigeria (African: YRI) and from the 60 CEPH-Utah residents with ancestry from northern and western Europe (European: CEU) populations were obtained from the International HapMap Project [30]. Gene expression data from the profiling of Epstein-Barr virus (EBV)-transformed lymphoblastoid cell lines from the individuals in each CEU and YRI HapMap population [33] were obtained from the National Center for Biotechnology Information Gene Expression Omnibus (GEO) database under accession number GSE10824. Figure 1 illustrates that using this model, TReQTLs can be identified which are associated with the downstream targets of TRs. The genetic variation attributed to the association are imbedded, and therefore discovered in the network of regulatory pathways that govern the co-regulation behind the phenotypic trait. The TReQTLs may be within a single gene (*cis*), spread across several genes (*trans*) or located in regions of unknown biological function. In addition, the case may be that several TReQTLs for the DSTs of TRs may share the same variants or portions of the same variants. To investigate the regulatory component of TReQTLs, we first sort out to determine if two populations (YRI and CEU) had shared or varied signaling transduction mechanisms robust enough for a more refined association analysis.

### Downstream Targets of Transcript-regulators

**Figure 2:**
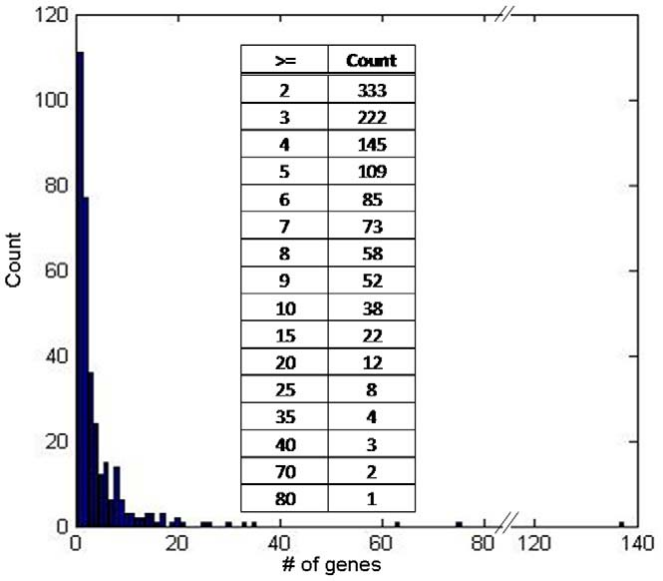
Distribution of the number of genes as downstream targets (DSTs) of transcript-regulators. The x-axis is the # of genes as DSTs and the y-axis is the count. The table inset is a summary of the frequency distribution for the count of the DSTs (two or more) per TR.

Using the TRANSFAC [31], [32] and TRANPATH [34] databases of components of signal transduction and regulatory pathways respectively, 2,743 TRs were mapped from the approximately 9,000 probe sets on the Affymetrix Human HG-Focus Target GeneChip Array, 1,438 signaling pathways were identified as comprised of at least one of the TRs and 78 TRs were mapped to one or more pathways. As shown in Figure 2, 333 TRs were determined to have two or more DSTs. These regulate 1,931 DSTs. The TRs consist of transfactors (TFs), cofactors, complexes and miRNAs. Three TFs, all stimulating proteins (Sp), regulate more than 60 DSTs. The median value for the TR DSTs is 3 and the mean is 5.8.

### Significant Signaling Transcript-regulators of Individual Populations

To determine the extent of co-regulation of genes within CEU and YRI populations, the correlation of the gene expression of pairs of the genes which are DSTs of TRs was assessed. Significance (*p*-value<or≈0.05) was determined from a non-parametric distribution of correlation scores obtained from 10,000 random cases. The correlation score is the sum of the squares of the Pearson correlations (*r*) among all pairs of genes determined to be DSTs of a TR (see the Materials and Methods section for more detail). For illustrative purposes, Table 1 reports the significance of the correlation of DSTs of only those TRs where disease-causing SNPs are located in the TR target site. The disease-causing SNPs were obtained from the NHGRI GWAS Catalog (www.genome.gov/gwastudies) and mapped to the TRANSFAC position weight matrix consensus sequence for the binding of at least one of the DSTs of the TR. Interferon-stimulated gene factor 3 (ISGF3), X-box binding protein 1 (XBP1) and hepatocyte nuclear factor 4-alpha (HNF4-α) are significant in the CEU and YRI populations. Signal transducers and activators of transcription −1 (STAT1), activating transcription factor 1 (ATF1) and peroxisome proliferator activating receptor gamma (PPAR-γ) are significant in YRI only whereas upstream transcription factor 1 (USF1), the Sp1∶Sp3 complex and the retinoid X receptor alpha (RXR-α):PPAR-γ complex are significant in CEU only.

**Table 1:** Co-regulation of DSTs of TRs where disease-causing SNPs are located in the TR binding site of at least one of the TR DSTs.

TR ID	TR Symbol	# of DSTs	CEU GCS	CEU p-value	YRI GCS	YRI p-value
T00428	ISGF-3	3	0.796	0.0055	0.328	0.0532
T00221	E2F:DP	7	1.374	0.3006	1.463	0.0629
T00902	XBP-1	2	0.565	0.0009	0.533	0.0001
T09484	NF-E2p45	4	1.148	0.0073	0.427	0.1163
T09998	c-Myc	3	0.541	0.0276	0.284	0.0821
T01804	NF-YA	13	6.222	0.0766	4.749	0.0482
T04759	STAT1	11	2.076	0.9201	3.452	0.0482
T09328	usf1	6	1.769	0.0268	0.607	0.4227
T10359	sp1∶sp3	2	0.252	0.0316	0.064	0.2137
T00167	ATF-2-xbb4	3	0.078	0.7219	0.073	0.6045
T03828	HNF-4alpha	12	6.445	0.0187	4.570	0.0222
T04870	MafG	2	0.284	0.0250	0.300	0.0062
T00968	ATF-1	4	0.544	0.1565	0.564	0.0459
T05351	PPARgamma	2	0.193	0.0660	0.184	0.0339
T08618	RXR-alpha:PPARgamma	2	0.237	0.0395	0.006	0.7065

GCS – Group correlation score. The disease-causing SNPs were obtained from the NHGRI GWAS Catalog (Available at: www.genome.gov/gwastudies. Accessed 3/3/2010) with selected SNP-trait associations limited to those with *p*-values<1×10^−5^.

### Genome-wide Analysis for Transcript-regulator Expression Quantitative Trait Loci

**Figure 3:**
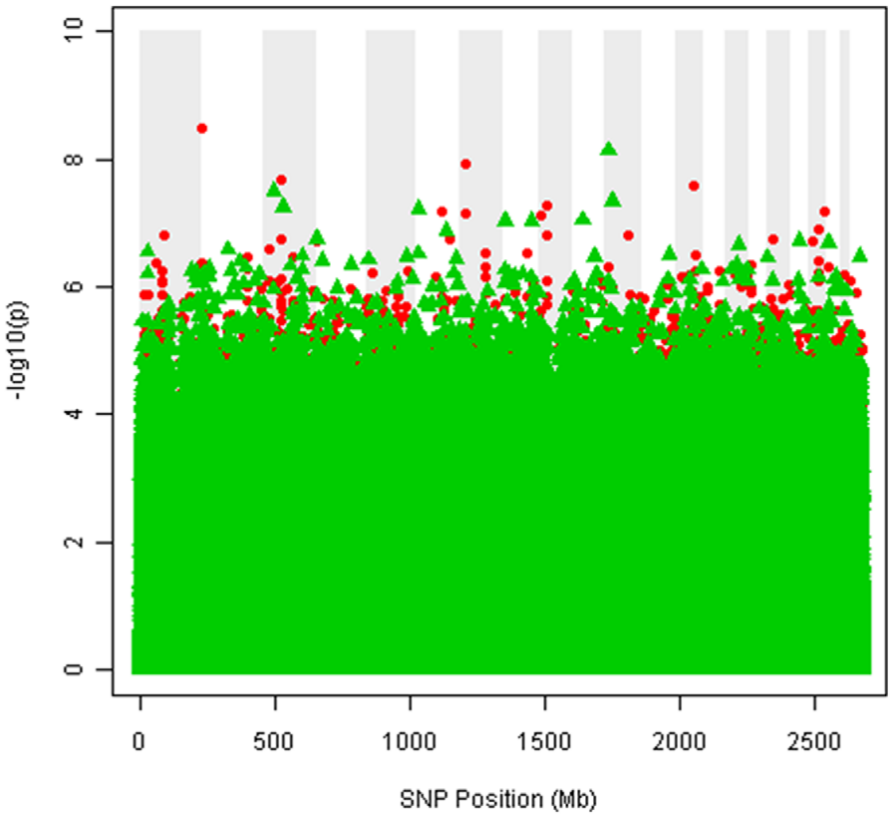
Manhattan plot displaying distribution of TReQTLs. The x-axis is the relative position of the SNPs across the genome in Mb. The chromosomes are illustrated by alternating shaded and unshaded sections of the plot. The order of the chromosomes is from #1 to #22 from left to right. The y-axis represents the –log_10_ *p*-value of the SNP association with the gene expression of DSTs of the TRs. *p*-values of SNPs from CEU are denoted as red circles, *p*-values of SNPs from YRI are denoted as green triangles. For visualization purposes, only SNPs having a *p*-value<0.002 in any of the 333 TRs are plotted.

To search for eQTLs that are tied to genes which are co-regulated in a given population, a multivariate linear regression was used to model the gene expression of the DSTs of TRs as response variables and the genotypes of SNPs as the predictor variables. The analysis was restricted to i) the 333 TRs which were found in the TRANSFAC database to have two or more DSTs (1,931 of the 8,399 unique UniGene transcripts represented by probe sets on the microarrays) and ii) to approximately 1.5 million SNPs on the autosomal chromosomes that passed the filtering criteria (see Materials and Methods) and were in common between CEU and YRI (common set), the 416,160 SNPs among the 1.5 million common set at a minor allele frequency (MAF)> = 0.05 and linkage disequilibrium (LD) *r* ^2^>0.5 (tag set) and the 184,616 independent (tag) SNPs which are within 1 kb of a gene (*cis*-acting set). For multiple testing correction, we used the 6.1×10^7^ *p*-values from the *cis*-acting set to control the false discovery rate (FDR = 45%). For the three TRs with 60 or more DSTs, the modified F-statistic [9], [35], [36] was used to obtain the nominal *p*-value for the TReQTL. A preliminary analysis considered a nominal *p*-value less than 1×10^−6^ for detecting TReQTLs. Although this cut-off is extremely high and subject to many false positives, we were initially interested in the overall robustness of the method to screen for putative associations. As illustrated in Figure 3, the TReQTLs for the CEU and YRI populations are widely different with 234 and 154 SNPs detected in CEU and YRI respectively. These represent 36 tag SNPs in CEU and 39 in YRI affecting the DSTs of 25 and 36 TRs respectively (Supplemental Materials Table S1). None of the TReQTL SNPs in the two populations overlap. At an FDR of 45%, two *cis*-acting tag SNPs (one in each population) are considered TReQTLs. In CEU, the SNP (rs16858621) in the pecanex-like 2 (*Pcnxl2*) gene was highly associated with the DSTs of the cAMP responsive element modulator (CREM) transfactor whereas in YRI, the SNP (rs16909324) was linked to the targets of miRNA hsa-miR-125a.

**Figure 4:**
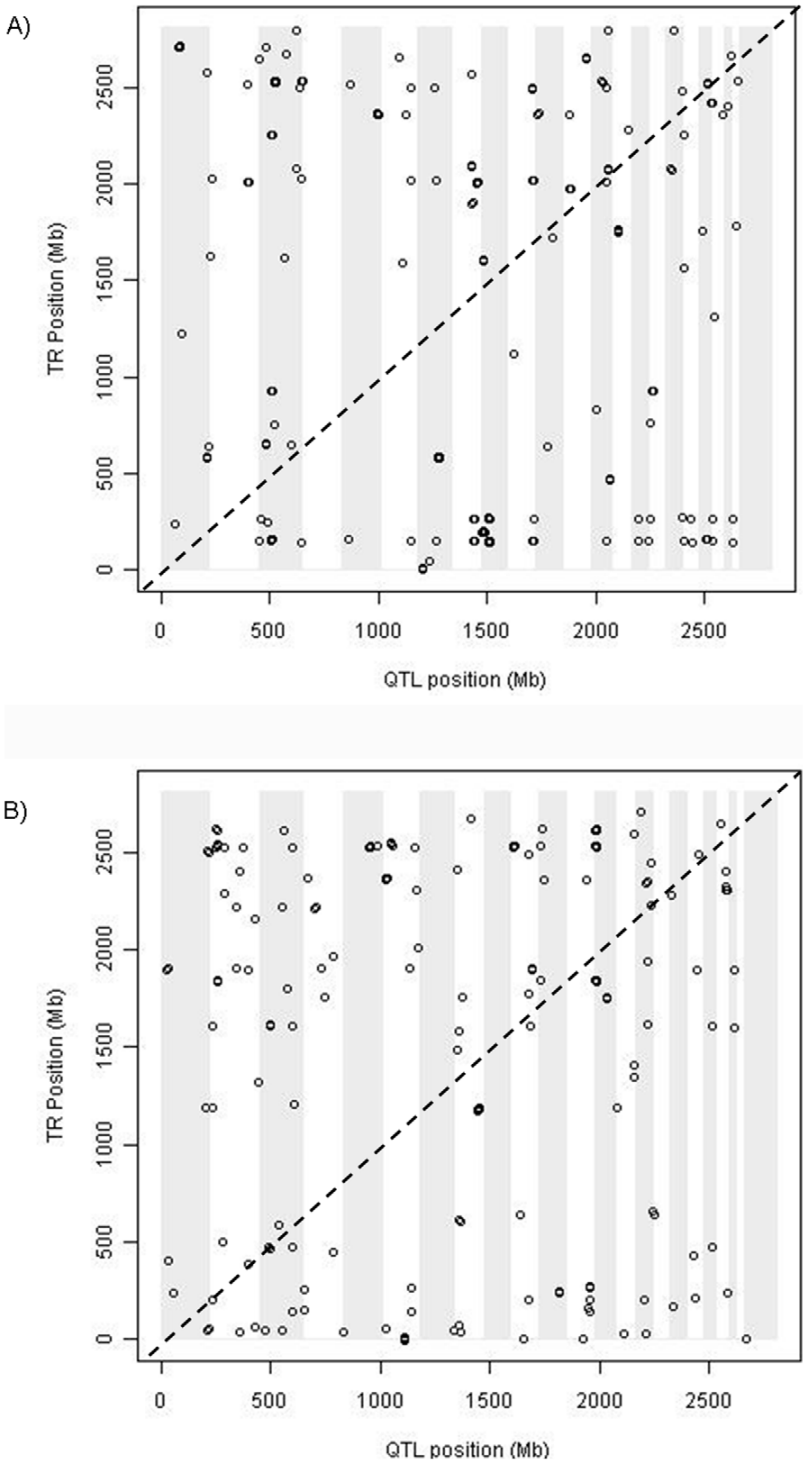
Dot plot of significant TReQTLs. A) CEU B) YRI. Each circle represents a TReQTL SNP with a *p*-value<1×10^−6^. The x-axis is the relative position of the TReQTL SNPs across the genome in Mb. The chromosomes are illustrated by alternating shaded and unshaded sections of the plot. The order of the chromosomes is from #1 to #22 from left to right. The y-axis is the relative position of the TR across the genome in Mb. The order of the chromosomes is from #1 to #22 from bottom to top. The points were jittered to enhance the display of TReQTLs in close proximity. TReQTLs near the diagonal line have the potential to be *cis*-regulated.

As shown in Figure 4, there are a few cases where the SNPs are mapped relative to a TR (i.e.<2 Mb). In CEU, the DSTs of TRs alpha-CBF (T00081) and ENKTF-1 (T00255) possessed 13 and 4 TReQTL SNPs respectively but are not displayed as these DST genes have not been characterized and hence, have no genomic location. Four tag SNPs were associated with the DSTs of the HIF2A:arnt complex (T10852) in CEU whereas 2 tag SNPs were associated with the DSTs of miRNA hsa-miR-125a (T09819) in YRI. Interestingly, in CEU, one tag SNP (rs16858621) was associated (*p*-value<5×10^−7^) with the DSTs of miRNA has-miR-15a (T09712) and TF CREM (T01803) both of which regulate *Ccnd1* [37], [38]. Thus, presumably, this represents a case where a SNP may affect a master regulator that controls not only a TF but a miRNA as well both of which share the role of regulating a common gene. In YRI, there are several cases where a tag SNP is associated with the DSTs of more than one TR. Although several sets of DSTs of TRs were found to have a fair number of significant tag SNPs mapped to them, two miRNAs (hsa-let-7e (T09710) in CEU and hsa-miR-200a (T09837) in YRI) have hotspots (SNPs in a region affecting multiple transcripts [15]) associated with the variation of expression of their DSTs according to the genotypes at the alleles. When restricting the comparison of the populations to the ∼1.5 million SNPs in common, several of the TReQTL overlapped between CEU and YRI. The expression of the DSTs of 24 TRs was associated with SNPs in both populations (Table 2). These were SNPs mapped within or in proximity to genes involved in transcription regulation, cell communication, transport, kinase activity, growth and development. Interestingly, several of the TReQTL SNPs in CEU are mapped to pseudogenes.

**Table 2:** TRs in common between CEU and YRI TReQTLs.

TR ID	TR Symbol	CEU SNP	Gene ID CEU SNP	Gene Symbol CEU SNP	YRI SNP	Gene ID YRI SNP	Gene Symbol YRI SNP
T00250	Elk-1	rs9838549	131185	LOC131185	rs965676	6638	SNRPN
T00255	ENKTF-1	rs10510093	2263	FGFR2	rs6864839	4488	MSX2
T00498	alpha-enolase	rs1029741	54543	TOMM7	rs12358485	359779	MRPS35P3
T00902	XBP-1	rs11686328	129563	DIS3L2	rs6111734	27131	SNX5
T00910	YB-1	rs17586344	1956	EGFR	rs11120212	100505832	LOC100505832
T01814	pax6-isoform5a	rs3212243	11035	RIPK3	rs10755971	137902	PXDNL
T01931	RelB	rs9610774	29775	CARD10	rs289838	9111	NMI
T02689	GATA-6	rs13345832	55769	ZNF83	rs2937889	57509	MTUS1
T04870	MafG	rs1153303	150000	ABCC13	rs12691592	53353	LRP1B
T04953	TFIIIA	rs11692860	729009	FTH1P20	rs7755681	5071	PARK2
T04959	GKLF-isoform1	rs9484664	100420742	LOC100420742	rs16848653	55137	FIGN
T04996	ZBP89	rs6691852	467	ATF3	rs6549604	5067	CNTN3
T05324	LXR-alpha:RXR-alpha	rs11157248	6955	TRA-alpha	rs7072859	2894	GRID1
T06135	p63gamma	rs6670238	51018	RRP15	rs1558561	9717	SEC14L5
T08465	C/EBPalpha	rs9068	220988	HNRNPA3	rs6570819	23328	SASH1
T08618	RXR-alpha:PPARgamma	rs1331584	150928	PTMAP5	rs4596085	11280	SCN11A
T09159	pitx2a	rs1983600	9742	IFT140	rs6966461	154664	ABCA13
T10331	NRF-1	rs7272098	6238	RRBP1	rs1347038	2043	EPHA4
T10852	HIF2A:arnt	rs2741270	248	ALPI	rs28740902	4487	MSX1
T11264	CP2	rs1020344	100130101	LOC100130101	rs3819726	4121	MAN1A1
T13796	TLS	rs10143078	55333	SYNJ2BP	rs870181	55275	VPS53
T14942	hsa-miR-181b	rs17543080	392285	LOC392285	rs10797531	148641	SLC35F3
T15206	N-Myc	rs2268943	4070	TACSTD2	rs1181164	148979	GLIS1
T15913	RXR-alpha:NR1B1	rs1855625	643954	RPSAP43	rs17238405	4734	NEDD4

### Cohesive TReQTL Biological Process Subtrees Reveal Descriptive Molecular Events

**Figure 5:**
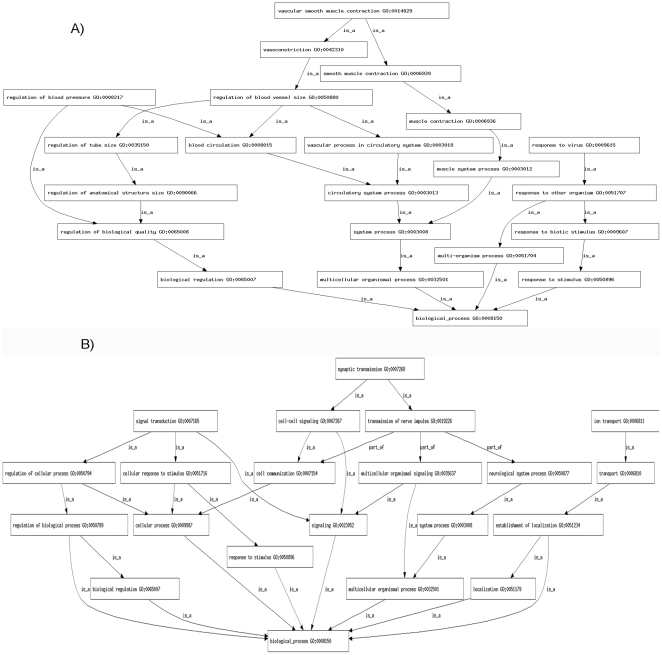
TReQTL Gene Ontology (GO) biological process subtrees. A) Based on the GO biological processes from the gene that the YRI TReQTL SNP rs12258754 map to and those of the DSTs of activating transcription factor 3 (Atf3) and of Atf3 itself. B) Based on the GO biological processes from the gene that the CEU TReQTL SNP rs10976413 map to and those of the DSTs of miRNAs hsa-mir-181b-1 (MI0000270) and hsa-mir-181b-2 (MI0000683).

Each TReQTL is comprised of a SNP, a TR and a set of DSTs. Each constituent is associated with a gene. We mapped the constituents, except for cases where the TR is a miRNAs, to genes and then determined the GO biological process term each was annotated to. The collection of terms was then used to construct a GO biological process subtree. All ancestors of a term were included in the subtree. Our adjusted cohesion score (ACS) is an ad hoc way to i) measure the amount of connectivity between terms, ii) account for the significance of the TReQTL and iii) consider the average number of paths per term. As listed in Table 3, the top ranked TReQTLs have the more cohesive subtrees and are more descriptive with respect to the term with most paths associated with it (The full list is in supplemental material Table S2 (CEU) Table S3 (YRI)). These associations within the subtree can lead to new insight into the possible role of the TReQTL SNP in the pathophysiology of diseases. For instance, in YRI, the rs12258754 allelic variant is associated with the DSTs of activating transcription factor 3 (Atf3) and produced a subtree with vascular smooth muscle cell (VSMC) contraction as the granular biological process node (Figure 5A). Interestingly, in CEU, four SNPs associated with the variation of expression for the DSTs of miRNAs hsa-mir-181b-1 (MI0000270) and hsa-mir-181b-2 (MI0000683) are mapped to the peptidyl-prolyl cis-trans isomerase A-like pseudo-gene and generated a subtree with synaptic transmission as the most descriptive biological process term (Figure 5B).

**Table 3:** TReQTL GO subtree cohesiveness.

							CEU				
TR ID	TR Symbol	SNP	p-value	ACS	# of GO Terms	# of Edges	# of Paths	Ave. # of Paths per GO Term	Max. # of Paths for Term	ID of GO Term with Max. # of Paths	GO Term
T00902	XBP-1	rs12664788	0.000000679	13.159	4	4	5	1.25	2	GO:0006955	immune response
T14942	hsa-miR-181b	rs10976413	7.63E-08	3.592	21	31	43	2.05	6	GO:0007268	synaptic transmission
T15286	E2F-1:DP-1	rs1382606	0.000000159	2.210	11	15	29	2.64	7	GO:0000080	G1 phase of mitotic cell cycle
T05444	RFX5:RFXAP:RFXANK	rs6940715	0.000000178	1.544	32	41	74	2.31	10	GO:0006281	DNA repair
T00902	XBP-1	rs12578202	0.000000955	0.788	38	54	113	2.97	15	GO:0001525	angiogenesis

							YRI				
T01313	ATF-3	rs12258754	0.000000331	5.516	22	26	32	1.45	4	GO:0014829	vascular smooth muscle contraction
T00902	XBP-1	rs6111734	0.000000199	3.619	21	27	42	2.00	5	GO:0015031	protein transport
T10747	MTF-1	rs12691592	0.00000071	3.275	21	26	41	1.95	5	GO:0006898	receptor-mediated endocytosis
T14942	hsa-miR-181b	rs10797531	0.000000597	3.141	21	31	43	2.05	6	GO:0007268	synaptic transmission
T10911	lmx1b	rs6681714	0.000000703	1.979	29	41	66	2.28	8	GO:0006468	protein phosphorylation

The top 5 TReQTL SNP for each transcript-regulator (TR) is listed for each population. The full list is in supplemental material Table S2 (CEU) Table S3 (YRI). ACS – Adjusted cohesive score.

### TReQTL interaction network

**Figure 6:**
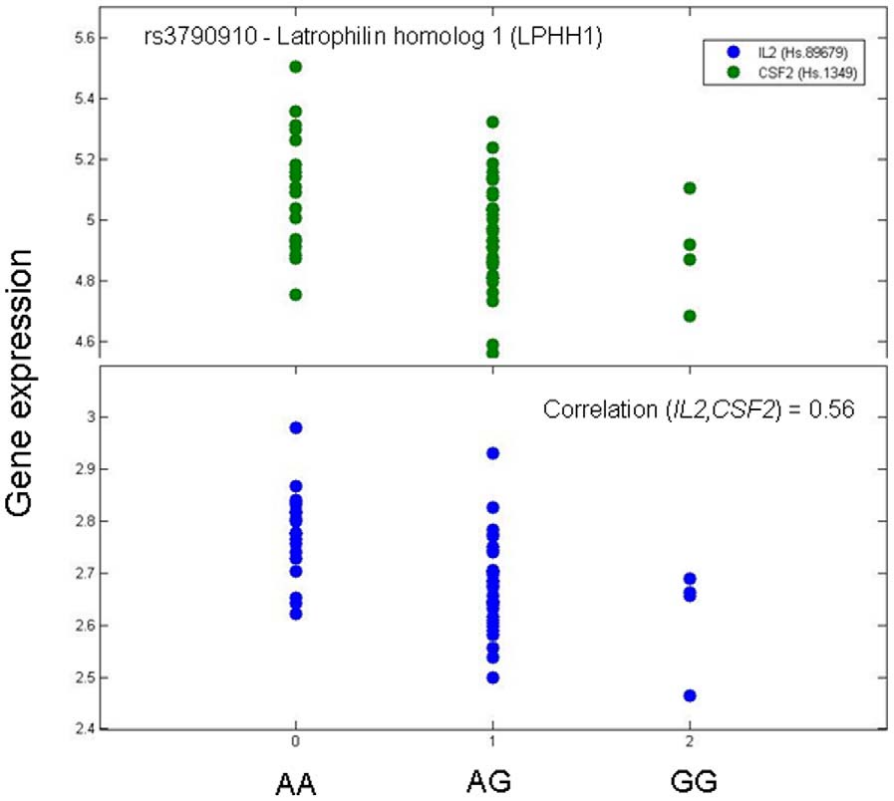
Scatter plot of differential expression of the DSTs of Foxp3. The x-axis is the genotype for SNP rs3790904 - Latrophilin homolog 1 (*Lphh1*/*Lphn2*). The SNP genotype is also coded as number of minor alleles. The y-axis is the log_ 2_ gene expression. The green dots are the expression from colony stimulating factor 2 (*Csf2*) and the blue dots are the expression from interleukin 2 (Il2). The Pearson correlation of the expression from *Csf2* and *Il2* is +0.56.

**Figure 7:**
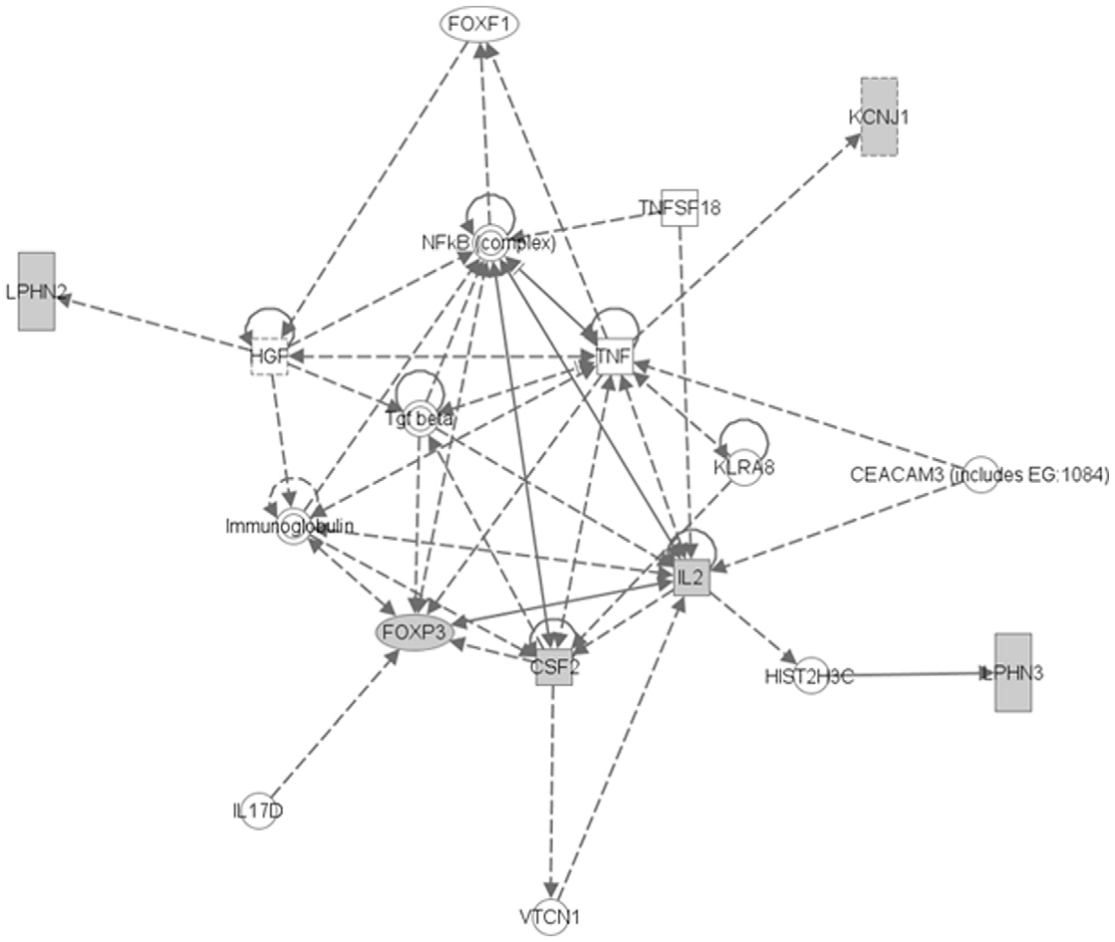
Foxp3 TReQTL network. The interaction network was generated by Ingenuity Pathway Analysis (IPA) software. Based on the IPA curated knowledgebase dashed lines represent indirect interactions and solid lines denote direct interactions. The arrow represents the process of acting on a target. Vertical rectangles are G-protein couple receptors, ovals are transcription regulators, squares are cytokines, double circles are complexs/groups and single circles are other types of biological molecules. Shaded nodes represent genes of molecules from the TReQTL for Foxp3 (those that the SNPs map to, the DSTs and the TR).

Many of the variants map to Online Mendelian Inheritance in Man (OMIM) associated disease genes (data not shown). For instance, one TReQTL in the CEU population is associated with the DSTs of the X-linked breast cancer suppressor gene *Foxp3* (T04280) transfactor [39]. *Foxp3* belongs to the Forkhead box family of genes, is located on chromosome X and is essential for the production and normal function of regulatory T-cells. As shown in Figure 6, interleukin 2 (*Il2*) and colony stimulating factor 2 (*CSF2*), the DSTs of Foxp3, are two cytokines whose gene expression co-regulation (correlation = 0.56) is significantly associated with the variants of tag SNP rs3790904 (*p*-value = 8.1 10^−7^) which maps to the latrophilin homolog 1 (*Lphh1*/*Lphn2*) G-protein couple receptor (GPCR) gene. This association is not significant in YRI (*p*-value = 0.89). Other significant SNPs in CEU that are linked to the DST of Foxp3 map to an additional GPCR gene (*Lphn3*) as well as a membrane ion channel (*KCNJ1*), a phosphatidic acid phosphatase type 2 enzyme (*Ppapdc1a*) and an uncharacterized gene. An interaction network of Foxp3, its DSTs and genes that the TReQTLs map to revealed tumor necrosis factor (TNF) and NF-kB as central hubs along with carcinoembryonic antigen-related cell adhesion molecule 3 (CEACAM3), Tgf-beta, and hepatocyte growth factor (HGF) as connectors (Figure 7). Interesting enough, negative regulation of NF-kappaB TF activity, cAMP response element-binding (CREB) activity and T-cell cytokine production/positive regulation of regulatory T-cell differentiation are biological processes within the Foxp3 TReQTL subtree that are highly connected (Data not shown).

### Over-representation of the Foxp3 TReQTL SNPs in Genomic Regions

SNPs in multi-species/evolutionary conserved regions (ECRs) may imply similarity of function across species [40] and those within splice junctions (SJs) or splicing enhancers may play a role in gene expression regulation through exon splicing [21], [41] . In other words, genetic variants that are related to gene expression differences between populations are more likely to be in genomic regions conserved across species and/or possibly involved in regulating transcription by altering splice forms of transcript messages. As an anecdotal example, we used 472 SNPs from the union of the Foxp3 TReQTL SNPs between CEU (n = 233) and YRI (n = 239) with a more liberal nominal *p*-value threshold <1×10^−4^ to determine over-representation within ECRs and SJs. As displayed in Table 4, using SNP-set enrichment analysis [42], the SNPs are significantly over-represented in 5-way ECRs for CEU (*p*-value = 0.006) but not YRI (*p*-value = 0.9) and enriched in SJs for YRI (*p*-value = 1×10^−4^) but not CEU (*p*-value = 0.9). Fisher exact tests confirm significance of the over-representation of these TReQTL SNPs in 5-way ECRs for CEU (*p*-value = 0.0079) and in SJs for YRI (*p*-value = 0.0001).

**Table 4:** Over-representation of TReQTL SNPs in genomic regions.

Genomic Region	CEU	YRI
5-way Conservation	0.006	0.900
17-way Conservation	0.071	0.870
Splice Junctions	0.893	1.0E-04

10 K permutations of 472 SNPs with a *p*-value<1×10^−4^ in either CEU or YRI.

## Discussion

Genetic and transcriptional variations are important key factors in the evolution of biology and the dispensation of diseases. Single nucleotide polymorphisms (SNPs) are one type of DNA sequence alteration that is commonly used as a marker for tracking genetic variation. The allelic frequency of a SNP at a given locus can vary between populations and the genotype may code for a SNP that results in a particular phenotype, trait or disease [43], [44], [45], [46]. Within populations and under certain biological conditions genes are coordinately regulated by transcript-regulators (TRs) such as transcription factors (TFs), cofactors, complexes of TFs and miRNAs (Table 1). These co-expressed genes often times share biological functions and work in concert to mediate cellular events such as biological processes and molecular pathways. Although it has been shown that TFs do not harbor *trans*-acting variants [11], coupling coordinately regulated genes as a quantitative trait for a loci (eQTL) with the genotype of SNPs as a genome-wide association study (GWAS) can presumably help to elucidate variation in gene expression (TReQTLs) on a genomic and systems biology scale that code for particular phenotypes and complex diseases [9].

Tailoring the GWAS eQTL analysis by considering genes with coordinated expression is of added value to reveal master regulators of transcriptional genetic variation (Figure 1). We used a multivariate linear regression with the gene expression of known downstream targets (DSTs) of TRs (Figure 2) as the response variable and individual SNPs as predictor variables to identify TReQTLs in European (CEU) and African (YRI) HapMap populations. At a nominal *p*-value threshold of <1×10^−6^ we discovered 234 SNPs in CEU and 154 in YRI as putative TReQTLs (Figure 4). These represent 36 and 39 independent (tag) SNPs in CEU and YRI affecting the DSTs of 25 and 36 TRs respectively. Two SNPs (one in each population) are *cis*-acting TReQTLs (within 1 kb of a gene) at a false discovery rate (FDR) of 45%. One of them, a SNP in the pecanex-like 2 (*Pcnxl2*) gene was found in CEU to be highly associated with the DSTs of the cAMP responsive element modulator (CREM) transfactor whereas in the YRI dataset, a SNP was linked to the DSTs of miRNA hsa-miR-125a. Although the FDR may seem abnormally high and one would expect at least one if not both of the TReQTLs to be false positives, it can be misleading as others have demonstrated that adjusting for biases which arise from correlations in eQTL analysis is a major challenge and a substantial overestimation of the number of false positives [47], [48], [49].

Interestingly enough, the gene expression of the DSTs of 24 TRs was associated with SNPs (albeit different ones) in both populations (Table 2) but the majority differed (Figures 3 and 4). The overlap in the TReQTLs probably reflects the ubiquity of certain basic biological processes such as transcription regulation, cell communication, transport, kinase activity, growth and development. On the otherhand, one TReQTL tag SNP (rs3790904) in the CEU population is associated (*p*-value = 8.1×10^−7^) with the DSTs of the X-linked breast cancer suppressor gene *Foxp3* (Figure 6) but is not significant in YRI (*p*-value = 0.89). The interaction network of the Foxp3 TReQTL in CEU revealed that tumor necrosis factor (TNF), NF-kappaB and variants in G-protein coupled receptors (GPCR) signaling may play a central role as communicators in Foxp3 functional regulation (Figure 7). Although the Foxp3 tumor suppressor is biologically relevant in the pathogenesis of breast cancer, some have shown that SNPs in the germline of the gene are not associated with the risk of the disease [50]. Our TReQTL analysis reveals other potentially interesting loci which might be causative in the etiology of complex diseases.

Another difference between the two populations based on the TReQTLs was the connectivity of the underlining Gene Ontology (GO) biological processes that the genes of the TReQTL represent (Figure 5). In CEU, several SNPs associated with the variation of expression for the DSTs of two miRNAs (hsa-mir-181b-1 (MI0000270) and hsa-mir-181b-2 (MI0000683)) are mapped to the peptidyl-prolyl cis-trans isomerase pseudo-gene and yields a subtree with synaptic transmission as the more cohesive descriptive GO term (Table 3). The activity of this enzyme has been suggested to be necessary for memory formation and may be involved in complex neurodegenerations such as Alzheimer's disease [51]. In YRI, a SNP (rs12258754) controlling the variation of expression for the DSTs of activating transcription factor 3 (Atf3) yielded a subtree with vascular smooth muscle cell (VSMC) contraction as the more descriptive GO term (Table 3). Although much is not currently known about the function of Atf3 in VSMCs [52], mutations in the actin, alpha 2 (*Acta2*) smooth muscle gene have been shown to result in a variety of vascular diseases [53]. Transcriptional networks such as these have been recently shown to be hubs with high connectivity and association with controlling higher-ordered biological function such as lipogenesis, lipid trafficking and surfactant homeostasis [54]. Our approach embraces this strategy by using the SNPs within the TReQTLs as an adjudicator for the identification of master regulators of these genetic networks. Although it is expected that a TR and its DSTs will share a common signaling pathway, what is not certain is that the SNP associated with the eQTL from the TR and DSTs will reside near or in a gene with biological functionality that forms a cohesive GO biological process subtree. Bear in mind that it is not known where the true regulating TR associated with a candidate TReQTL actually exerts its biological functionality and to date, there is no independent data set with gene expression and genotype calls from another sample of the YRI and CEU populations to replicate our results. However, once the genotype data from Idaghdour et al. [16] are made publicly available, we will be able to use it to determine if our TReQTLs can discern between Moroccan populations according to geographical locations, regional differences and ancestry. Furthermore, in depth functional analyses on TR targets will presumably shed light on these TReQTL regulatory networks and perhaps biologically confirm our results.

McCauley et al. [40] reported that SNPs in multi-species conserved sequences (MCS) are useful as markers linking to complex diseases. Recent evidence suggests that SNPs that influence alternative splicing are enriched within splice junctions (SJs) or disrupt splicing enhancers [21], [41]. Our analysis of Foxp3 TReQTLs revealed SNPs overrepresented within 5-way (human, mouse, chimp, rhesus monkey and dog) evolutionary conserved regions (ECRs) in CEU and in SJs of YRI defined by RNA-Seq mapping (Table 4). These results support the notion that genomics, genetics and transcriptomics play an intricate role in sustaining population diversity and structure [16]. It would be interesting to determine how environmental factors, population structure and geographical differences affect transcript abundance as a quantitative trait when co-regulation of gene expression is considered.

Although the identification of TReQTLs is useful for determining genetic variants regulating gene expression, there are limitations to the approach and guidelines with interpretation of the results. First, there is a paucity of information about the genes which TRs control. We restricted our analysis to only 333 TRs with two or more DSTs known at a given time to be regulated by TRs. This does not capture the full array of genetic variants which might contribute to the gene expression differences between the two populations. However, as advances in functional genomics leads to improved knowledge about gene regulation and biological function on a genome-wide scale, the discovery of TReQTLs should advance and be more informative. In addition, the study of the transcript-regulation of genes by miRNA is in its infancy and there is a small number of miRNAs known to regulate genes. Furthermore, our analysis only tested the association of a single SNP with sets of coordinately expressed genes. It is very likely that the variation in expression is due to the synergistic effect of two or more SNPs. In fact, there may be other mediators of complex diseases other than SNPs acting alone or symbiotically. Finally, our work relied on samples from immortalized lymphoblastoid cell lines (LCLs) and not from a disease state. Therefore, it is debatable whether or not the genetic associations of SNPs with gene expression in LCLs will carry over to tissue samples from organs [55]. However, there is some indication, albeit a paucity of evidence, that the DNA repair capacity of LCLs from breast cancer samples is significantly lower than control subjects [56], that tumor-infiltrating Foxp3+ regulatory T cells can distinguish between high-risk breast cancer patients and those at risk of a late relapse [57] and that a fraction of eQTLs derived from the analysis of UK Adult Twin registry LCLs gene expression and genotype data overlap with those identified in a HapMap population [47]. Despite the caveats noted above, the advantages of associating genetic markers such as SNPs to quantitative traits such as co-regulated genes is promising and of value as an additional strategy when investigating the role of a genetic variant and master regulators in the etiology of a complex diseases.

## Materials and Methods

### Genotype Data

Genotype data (phase-II, release 24, forward strand, non-redundant) from the 60 Yoruba in Ibadan, Nigeria (African: YRI) and from the 60 CEPH-Utah residents with ancestry from northern and western Europe (European: CEU) populations were obtained from the International HapMap Project [30]. SNPs with a call rate <95%, minor allele frequency (MAF)<0.05, or Hardy-Weinberg equilibrium [58] *p*-value<0.05 within each population separately were removed and we restricted our analysis to autosomal markers only. About 2 million SNPs in CEU and ∼2.2 million SNPs in YRI were retained after filtering. The approximately 1.5 million SNPs in common between the two populations after filtering (common set) were used for TReQTL preliminary analysis using a nominal *p*-value threshold of <1×10^−6^. To account for correlated SNPs, we used the **LRTag** approach [59], [60] with linkage disequilibrium (LD) correlation (*r* ^2^)>0.5 and MAF> = 0.05 to tag 416,160 independent SNPs (tag set) of the 1.5 million in the common set. In addition, for multiple testing correction, we used the *p*-values of the tag SNPs that are within 1 kb of a gene (*cis*-acting set) to obtain an FDR. For the SNP association portion of the study, we focused the sample set on the 60 CEU and 55 YRI individuals that had corresponding gene expression data.

### Microarray Gene Expression Data

Gene expression data from the profiling of Epstein-Barr virus (EBV)-transformed lymphoblastoid cell lines from the individuals in each CEU and YRI HapMap population were acquired from assaying total RNA on Affymetrix Human HG-Focus Target GeneChip Array [33] containing about 9,000 probe sets and representing about 8,600 UniGenes (NetAffx array annotation release 29, March 30, 2009 UniGene build 219). The data are available in the National Center for Biotechnology Information (NCBI) Gene Expression Omnibus (GEO) database [61], [62] under accession number GSE10824. The data were log_2_ transformed and normalized using the robust multi-array average method [63]. The gene expression data from probe sets that mapped to the same UniGene cluster were averaged resulting in 8,399 unique UniGene transcripts represented by probe sets on the array.

### RNA-Seq Data

We obtained raw sequence reads from the whole transcriptome sequencing of the CEU and YRI populations [64], [65]. These reads were mapped to the reference human genome (hg18, NCBI 36) using the **Bowtie** program [66]. Splice junctions (SJs) were mapped using the **Tophat** program [67]. Transcript abundance was calculated by the **Cufflinks** program [68] and normalized using the FPKM (Fragment Per Kilobase of Exon per Million reads) method [69]. At a nominal *p*-value<1×10^−4^ there are a total of 7,149 and 7,040 TReQTL SNPs in the CEU and YRI populations respectively. Each SNP was evaluated for its presence in SJs in each individual from both populations. The total number of SNPs in each population was calculated based on their presence or absence in any individual of the population.

### Signal Transduction Pathway Profiling

The Affymetrix GeneChip array probe sets were collapsed into approximately 8,600 UniGene transcript clusters using the March 30, 2009 release of UniGene (build 219). The gene expression data from probe sets that mapped to the same UniGene cluster were averaged resulting in 8,399 UniGene transcripts. The UniGene downstream targets (DSTs) of transcript-regulators (TRs: transcription factors (TFs), miRNAs, cofactors and complexes) were obtained from the March 26, 2010 release (version 2010.1) of the TRANSFAC® database [31], [32]. TRs were mapped to signaling pathways using the June 26, 2009 release (version 2009.2) of the TRANSPATH® database [34]. Significance of signal transduction pathway profiling was determined as previously described [70]. Briefly, for each population individually, significant TRs were based on a Group Correlation Score
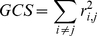
(1)defined as the sum of the squares of the Pearson correlations (*r*) among all pairs of genes *i* and *j* determined to be DSTs of the TR. Significant pathways made up of TRs are based on an Exclusive Group Correlation Score
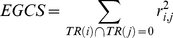
(2)defined as the sum of the squares of *r* over all pairs of genes *i* and *j* in a pathway that do not share any TR. This eliminates the contribution of the co-expression of DSTs that share TRs. The *p*-value for a score was determined from a non-parametric distribution of correlation scores obtained from random cases (*B* = 10,000 reshuffles of the genes) and the number of times (*n*) one of these permuted scores is greater than the observed correlation score. Thus, 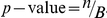 For both correlation scores, GCS and EGCS, this null hypothesis keeps the structure and overlap of all pathways fixed, but changes the identity of the genes.

### TReQTL Analysis

For each population and TR, we performed a genome-wide scan by regressing the log_2_ expression levels of the DSTs on each SNP genotype (*Z*) (coded 0, 1, and 2 representing the number of minor alleles) separately across the genome. The following multivariate linear regression (MVR) model was used
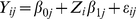
(3)where *Y_ij_* denotes the log_2_ expression levels of the DST *j (j = 1,…, m)* for a TR for subject *i* (*i = 1,…,n*), *m* is the number of DSTs of the TR, Z_i_ is a SNP genotype, å_ij_ is an error and ε*_i_* = (ε_i1,…_, ε_im_) follows a multivariate normal distribution with mean 0 and covariance Σ. To test for the null hypothesis of association between a SNP and a TR, we performed the likelihood ratio test for testing the null hypothesis H*_0_*: *β_11_* = … = *β_1m_* = 0, which follows a chi-square distribution with *m* degrees of freedom for *m<n*. Let the chi-square test statistic *D* = −2(ln(likelihood null model)–ln(likelihood full model)) where the null model is the MVR model without the genotypes corresponding to the SNP and the full model is the MVR model with the genotypes corresponding to the SNP. The *p*-value for each association of a SNP and set of DSTs for a TR was obtained from the distribution of *D*≈χ^2^ with degrees of freedom = *m*. For cases where *m*> = *n*, an approximate F-statistic [9], [35], [36] was used in order to avoid situations where the covariance matrix from the MVR model is not full rank. In these cases, the *p*-value for statistical significance of each association of a SNP and a set of DSTs for a TR was assessed by permuting the *n* rows and *n* columns of the F-statistic G (Gower's centered) matrix (1×10^6^ times) and determining the number of times one of these bootstrapped scores is greater than the observed score. We fit model (3) by regressing the DSTs of a TR on each SNP separately across the genome. For multiple testing correction, we used the 6.1×10^7^ *p*-values from the regression of the DSTs of the 333 TRs on the 184,616 independent (tag) SNPs that are within 1 kb of a gene (*cis*-acting set) to control the FDR [71] at 45%.

### SNP Set Enrichment Analysis

To determine whether a set of SNPs representing TReQTLs are enriched within the genome, a variation of gene set enrichment analysis [72] was used. Rather than enrich for SNPs within pathways as previously described [42], we test for enrichment of SNPs within particular genomic regions. The regions of interest are either evolutionary conserved regions (ECRs) or splice junctions (SJs). The rationale is that variants which are related to gene expression differences between populations are more likely to be in genomic regions conserved across species and\or possibly involved in regulating transcription by altering splice forms of transcript messages. The ECRs are from 5-way (human, mouse, chimp, rhesus monkey and dog) and 17-way (human, chimp, macaque, mouse, rat, rabbit, dog, cow, armadillo, elephant, tenrec, opossum, chicken, frog, zebrafish, Teraodon, and Fugu) conservation scores for the +/− 20 kbp flanking regions of the genes. Overlapping chromosomal location intervals for phastCon scores [73] were segmented and the highest conservation score within the interval was obtained. SJs were mapped using **Tophat** [67]. Transcript abundance was calculated by **Cufflinks** [68] and normalized using FPKM [69] (see the RNA-Seq methods section). For each TR, given a combined set *L* of SNPs associated with significant TReQTLs within the CEU or YRI population, their corresponding *p*-values and the +1 or −1 indication (flag) of the assignment of the SNP either within or not within genomic region set *S_i_*, an enrichment score (ES) is obtained by the ranking of the SNPs in ascending order (most significant to least significant), and then summing up the assignment flags. The ES is calculated by screening this list from the top to the bottom and increasing (decreasing) a running sum Kolmogorov–Smirnov-like statistic [72] when encountering or not encountering a SNP in a genomic region. A normalized ES (NES) is obtained by accounting for the size of the genomic region set *S_i_*. The *p*-value for enrichment was determined from a non-parametric distribution of NESs obtained from random cases (10,000 reshuffles of the assignment of the SNP (either within or not within a genomics region)) and the number of times one of these permuted scores is greater than the observed NES. Significance of enrichment was also confirmed using a two-tailed Fisher's exact (parametric) test.

### Ranking of the TReQTL SNPs

The ranking of TReQTL SNPs was performed by first measuring the cohesion of GO biological process terms in TReQTL *i*:
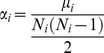
(4)where *N_i_* is the number of nodes (biological process terms) represented in TReQTL*_i_* and *μ*_i_ is the number of the edges between nodes. The edges were derived from the structure of GO subtree for each TReQTL*_i_* created from the biological process terms of the gene that the SNP maps to or is in close proximity, those of the TRs (excluding miRNAs) and the DST genes. The cohesion measure *α_i_* is then weighted by the *p*-value of TReQTL*_i_* to give a weighted rank. The weight is computed as −2log_10_(*p*-value). Finally, the number of paths and the number of biological process terms within the subtree were used to derive of the adjusted cohesion score
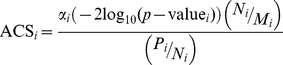
(5)where for the *i*th TReQTL, *M_i_* is the maximum number of paths of a biological process term and *P_i_* is the number of paths.

### Gene Interaction Network

Ingenuity Pathway Analysis (IPA) software version 8.8 and canonical pathway content version 3204 were used to build gene interactions from Foxp3, its DSTs (*CSF2* and *Il2*) and the significant TReQTL genes from CEU and mapped on chromosome 1 (*KCNJ1*, *LPHN2* and *LPHN3*).

## Supporting Information

Table S1 The TReQTLs for the CEU and YRI populations. Tab-delimited text file.
(TXT)

Table S2 Gene Ontology biological process subtrees from the CEU TReQTLs. Tab-delimited text file.
(TXT)

Table S3 Gene Ontology biological process subtrees from the YRI TReQTLs. Tab-delimited text file.
(TXT)
